# Safety Implications of Off-Label Medication Use in Athletes: A Narrative Review

**DOI:** 10.3390/medicines11080020

**Published:** 2024-11-15

**Authors:** Vítor Silva, Ricardo Madeira, João Joaquim, Cristiano Matos

**Affiliations:** 1 Unidade Local de Saúde (ULS) de Coimbra, 3004-561 Coimbra, Portugal; vahsilva@hotmail.com; 2 Polytechnic Institute of Coimbra, Coimbra Health School, Farmácia, 3046-854 Coimbra, Portugal; ricardom.99@hotmail.com (R.M.); jjj@estesc.ipc.pt (J.J.); 3 QLV Research Consulting, 3030-193 Coimbra, Portugal

**Keywords:** off-label use, performance enhancement, anabolic androgenic steroids, health risks

## Abstract

In recent years, the off-label use of medications in sports has increased significantly, primarily driven by psychological and social factors. Athletes frequently misuse drugs without adequate medical supervision, relying on unreliable sources of information, which leads to improper usage and serious health risks. This narrative review analyzes literature from PubMed^®^ (Medline), Scopus^®^, and Web of Science^®^ databases, focusing on studies up to December 2023, to examine the safety concerns related to off-label drug use in sports. The review presents an overview of the off-label use of pharmacological substances by athletes, focusing on both hormonal and non-hormonal drugs. Hormonal substances such as anabolic steroids and growth hormones, and non-hormonal agents like diuretics and β2-agonists, are frequently abused. These practices are associated with severe side effects, including infections, cardiovascular complications, hormonal imbalances, psychological disorders, dependence, and even cases of death. The study emphasizes the need for stronger regulation, public awareness initiatives, and preventive strategies to mitigate the health risks associated with this growing trend.

## 1. Introduction

In recent years, there has been an increase in the off-label use of certain medications [1]. Off-label use could be defined as “a situation where the medication is intentionally used for a clinical purpose not in accordance with the product’s marketing authorization” [2]. A drug officially approved for certain conditions or populations may be prescribed off-label based on clinical judgment, emerging research, or anecdotal evidence [3,4]. This approach offers flexibility in medical treatment, allowing for personalized care and innovative therapeutic options, especially when standard treatments are limited or ineffective [3]. However, off-label use differs from standard medical practices because it often lacks the rigorous clinical trials and comprehensive evidence that support approved uses [3,5]. Regulatory bodies, such as the Food and Drug Administration (FDA) or European Medicines Agency (EMA), typically do not oversee off-label applications directly. Instead, the responsibility for off-label prescribing falls entirely on the prescriber [6,7]. While regulatory authorities can issue warnings if safety concerns arise, the decision to prescribe off-label is largely left to the clinical judgment and discretion of healthcare professionals [7].

The use of medicines outside the marketing approval indication is a practice widespread across both professional and amateur athletes, including gym-goers and bodybuilders [8,9]. Whether in pursuit of enhanced performance, physical appearance, or faster recovery, athletes are increasingly utilizing medications under off-label indications, raising significant safety and ethical concerns [8,9]. The performance-enhancing drugs (PEDs) include substances that improve athletic performance by increasing endurance, strength, and/or focus. [1,10,11]. Most common examples are anabolic steroids, growth hormones, stimulants, and others that are often used outside their approved medical approval to gain a competitive edge [12]. PEDs are typically associated with potential for misuse, lack of evidence on long-term effects, and violations of anti-doping regulations [13]. Their use presents considerable risks, particularly in terms of their long-term health effects and fairness in competitions [14,15], including undermining the integrity of competitive environments [14,15].

The off-label use also allows athletes to take advantage of the pharmaceutical properties of drugs to achieve competitive advantage [16], as drugs intended to treat conditions like asthma, which are often repurposed to improve aerobic capacity [16,17]. The pursuit of this competitive advantage often occurs in a high-pressure environment where success can lead to significant financial and social rewards, driving athletes to seek any potential edge. The practice is, however, fraught with risks, such as development and side effects, ethical dilemmas, as well as the potential for sanctions in sporting activities where the use of the drug violates anti-doping regulations [18].

These substances are frequently administered orally or injectable, often in a cocktail of drugs to obtain a supposedly synergistic effect through complementary mechanisms of action [19,20]. Such practices, especially involving injectable administration, are typically conducted in non-aseptic environments, with drugs being shared among users, reused without proper sterilization, or stored improperly, significantly increasing the risk of infections and other health complications, such as adverse effects [19,20]. The sources from which these substances are obtained add to the complexity of the situation, as many individuals acquire these drugs from unregulated or unreliable suppliers, through non-medical channels such as fake online pharmacies, black market dealers, or through colleagues [21,22]. The lack of regulation and oversight in these transactions results in counterfeit or contaminated products circulating, posing significant health risks to users [20,23]. The consequences of off-label use can be severe, ranging from infections [14,24] or allergic reactions [7,14] to more serious conditions, including cardiovascular problems [25], hormonal imbalances [26], psychological issues [27], dependence [24], social episodes of violence [28], and even death [8,29,30]. 

Due to these risks, access to these substances remains by prescription only [1]. However, while many of these substances are manufactured by legitimate pharmaceutical companies, there is a growing prevalence of smuggled and counterfeit drugs [20,31], which often result in adulterated or contaminated products containing incorrect dosages, different substances, harmful impurities, or the presence of toxic substances [20,23]. To address these issues, the European Union has implemented strict regulations to control the manufacturing, importation, and marketing of these substances, as outlined in Directive 2011/62/EU of the European Parliament and the Council of 8 June 2011 [32].

While these medications, both hormonal and non-hormonal, are approved for specific medical conditions, their off-label use by athletes introduces additional risks, particularly due to improper dosing, lack of medical supervision, and the use of these substances outside their intended therapeutic context. Hormonal agents, including anabolic steroids and growth hormones, influence the body’s endocrinal system and are associated with dangerous hormonal imbalances and various side effects, including hypertension, changes in metabolism, and even increased cancer [33]. For instance, anabolic steroids can lead to hypertension [1,9] liver damage [29], and reproductive health issues [34], while growth hormone misuse can cause joint pain, insulin resistance, and an increased risk of cancer [30]. In contrast, non-hormonal medications, such as benzodiazepines and β2-agonists, can cause dependence, psychological effects, and cardiovascular complications [33]. 

This review aims to present an overview of the off-label use of pharmacological substances by athletes, which includes the use of hormonal and non-hormonal substances to enhance performance, better physical appearance, and rapid recovery from injuries.

## 2. Materials and Methods

This study follows a narrative literature review methodology to evaluate the off-label use of medications by athletes in sports practice. A comprehensive bibliographic search was conducted to gather relevant scientific articles in PubMed^®^ (Medline), Scopus^®^, and Web of Science^®^.

### 2.1. Search Strategy

A combination of specific search terms tailored to capture articles discussing the off-label use of medications in sports was applied. These terms included “off-label drug use”, “performance-enhancing drugs”, “athletes”, “gym-goers”, “steroids”, “growth hormone” and “non-prescribed substances”. We also employed Boolean operators such as AND, OR, and NOT to refine the search results and ensure that we captured the widest range of relevant studies without irrelevant overlaps.

### 2.2. Inclusion and Exclusion Criteria

We included articles based on the following criteria: they had to be published in Portuguese or English, covering studies from inception until December 2023. The articles needed to specifically address the off-label use of prescription medications in sports, particularly those focusing on athletes, gym-goers, or bodybuilders. We excluded articles that did not directly discuss the off-label use of medications in these contexts or were case reports, letters to editors, or opinion pieces lacking substantial empirical data.

### 2.3. Data Collection and Analysis

The search was conducted in November 2023, and we exported the articles that met the inclusion criteria into a reference management tool (Mendeley^®^). The final dataset of articles was qualitatively analyzed to identify recurring themes, the types of off-label drugs used, associated risks, and their implications for athletes’ health. The analysis aimed to identify the most commonly used off-label substances, such as anabolic steroids, growth hormones, and β2-agonists, and their documented health risks. Furthermore, we considered the social and ethical dimensions of off-label drug use in sports.

## 3. Results

Substances used off-label in sports practice vary with the type of athlete, type of sport, and the desired outcome. Those substances include both hormonal and non-hormonal molecules, with their therapeutic use, off-label use, and the risks associated with off-label use in sports practice described in Table 1 and Table 2, detailing their therapeutic use, off-label use, and associated risks. However, a notable concern in sports is the common practice of using multiple substances simultaneously, often without proper medical guidance. This practice is particularly risky as it raises the odds that drugs will interact in adverse ways. This can allow the combined effects of two or more drugs to amplify, and that produces compounded risks. As a result, the combination of anabolic steroids with erythropoietin may increase the risk of such cardiovascular events as thrombosis or myocardial infarction [35]. Diuretics may also be used with performance-enhancing drugs, leading to severe dehydration and electrolyte imbalance, increasing the risk of cardiac arrest, or other life-threatening conditions [18,36]. The compounded effects of this are especially concerning because they can go undetected until severe and/or irreversible harm has taken place. This has important implications for sports, where the wide dissemination, misuse, and off-label use of polypharmacy raises serious concerns for athlete health.

During use, consumers use substances concomitantly, sometimes mixing multiple substances to achieve an assumed synergistic effect or to counteract possible adverse effects [19,20]. The most used substances are primarily hormonal, such as testosterone, growth hormone, insulin, erythropoietin, and thyroid hormone [37].

Erythropoietin (EPO) is used in safe doses to control erythropoiesis [38], increase oxygen transport capacity by red blood cells [38], and treat anemia resulting from chemotherapy or chronic kidney disease [39]. In sports, it is used to increase hemoglobin levels, subsequently increasing oxygen in the blood, and improving performance in endurance training and competitions [38]. Despite being prohibited by the World Anti-Doping Agency, athletes continue to use EPO to boost their performance in endurance sports such as running, cycling, and skiing [40]. In healthy non-athlete subjects, erythropoietin administration prolongs submaximal exercise performance by about 54% independently of the approximately 12% increase in VO_2_max [41].

hCG is commonly used as an ovulation stimulant and in gonadotropin treatments [42]. It plays an important role in the development of the placenta and fetal growth of spermatogenesis and acts as a tumor marker in different types of cancer [42,43,44]. As off-label, hCG is used to increase muscle strength and boost testosterone levels [44]. It is particularly valued for its ability to stimulate endogenous testosterone production, making it a popular choice among athletes seeking to enhance performance and physical appearance [44]. When exogenous anabolic-androgenic steroids are introduced into the male body, inherent negative feedback loops shut down the body’s endogenous production of testosterone by shutting down the hypothalamic–pituitary–adrenal axis [37,45]. Among other things, this leads to testicular atrophy [1,9]. Commonly, HCG is used during and post-steroid cycles to maintain and restore testicular size, as well as normal testosterone production [45].

Growth hormone in controlled doses is used for treating male hypogonadism [46], inflammatory bowel disease [1], musculoskeletal conditions [46], neonatal hypoglycemia [1,46,47], and infertility [46]. In sports practice, it is sought to increase muscle strength [9,46,47], lean body mass [9,46,47], induce lipolysis [46,47], and accelerate recovery from soft tissue injuries [1,46,47].

**Table 1:** Hormonal substances used, therapeutic use, off-label use, and previously described AEs in off-label use in sports training.

Substance Name and Anatomical Therapeutic Chemical Code	Therapeutic Use	Off-Label Use in Sports Training	Previous Described AEs in Off-Label Use
Erythropoietin B03XA01	- Controls erythropoiesis [38]; - Increase oxygen transport capacity by red blood cells [38]; - Anemia resulting from chemotherapy or chronic kidney disease [39].	- Increase hemoglobin levels, subsequently increasing oxygen in the blood, improving performance in endurance training and competitions [38,48].	Nausea [38,49]; headache [38]; injection site infection [38]; blood clotting [38,50]; high risk of thrombosis [35,38]; myocardial infarction [38]; severe anemia [38]; inhibits apoptosis, which can lead to cancer [38,49].
Human Chorionic Gonadotropin (hCG) G03GA01	- Ovulation stimulants and gonadotropins [42]; - Development of the placenta and fetal growth [43]; - Spermatogenesis [44]; - Tumor marker [43].	- Increase muscle strength [44,48]; - Boost testosterone levels [44,48].	Suppression of the function of the hypothalamic–pituitary–adrenal axis [9]; decreasing endogenous testosterone production [51], leading to testicular atrophy [1,9]; erectile dysfunction and gynecomastia [1,9,20].
Human Growth Hormone H01AC01	- Male hypogonadism [46]; - Inflammatory bowel disease [1]; - Infertility treatment [46]; - Musculoskeletal conditions [46]; - Neonatal hypoglycemia [1,46,47].	- Increase lean body mass [9,46,47]; - Reduce body fat [46,47], induce lipolysis [46]; - Increase muscle strength [9,46,47]; - Accelerate recovery from soft tissue injuries [1,46,47,52].	Abdominal pain [1]; carpal tunnel syndrome [1]; flatulence [1]; facial edema [1]; headache [1,47]; nausea [1]; insulin resistance [20,53] and possible type 2 diabetes [1,20]; cardiac instability [20,53]; hypertension [20,46]; sleep apnea [47]; abnormal bone growth [53] [46,47]; muscle weakness [46]; fluid retention [46].
Insulin A10AB01	- Diabetes treatment [46].	- Increase glucose transport in the [46,54]; - Induce hyperinsulinemia (increase amino acid transport to muscles inhibiting protein breakdown) [20,55]; - Increase body mass [19].	Hypoglycemia (loss of consciousness, coma, and death) [38,56]; respiratory problems [54,56]; chest pain [56]; dehydration [54,56].
Oxandrolone A14AA08	- Promotes weight gain in patients after surgery or chronic infections [57]; - Used to counteract catabolic states [58]; - Treatment of bone pain associated with osteoporosis [59]; - Treatment of delayed puberty [60]; - Several burns [58,61].	- Increase muscle mass [62]; - Enhance recovery [62].	Liver toxicity [62]; hyperlipidemia [63]; cardiovascular strain, including hypertension and an increased risk of heart attack [64]; suppression of natural testosterone production [62].
Testosterone G03BA03	- Treatment of primary and secondary males; - Hypogonadism [1,9].	- Low energy [1]; - Increase muscle strength [9]; - Muscle growth [9]; - Weight loss [9] (dose-dependent) [20].	Alopecia [1]; hypertension [1,9]; renal retention (fluids) [9]; musculoskeletal [9]; immune [9]; dermatological [1,9]; suppresses the hypothalamic–pituitary–adrenal axis function [9]; decreasing endogenous testosterone production [51]; testicular atrophy [1,9], erectile dysfunction, and gynecomastia [1,9,20].
Thyroid Hormones H03AA01	- Primary and secondary hypothyroidism treatment [1].	- Fatigue [1,65]; - Lost fat mass and lean body mass [1,65]; - Depression [1]; - Cognitive and physical deficiency [1,66].	Iatrogenic thyrotoxicosis [1,67]; heart failure [1]; tremors [1]; weakness [1,67]; alopecia [1]; tachycardia [1,68].

Siebert et al. showed that the use of GH in athletes was not superior to placebo, concerning maximum aerobic capacity [47]. The use of GH promoted improvement in 3.8% of maximum anaerobic capacity in the Wingate cardiopulmonary stress test [47,69]. This improvement had a significant impact in a sporting competition environment and occurred independently of the gain in muscle mass [47].

Testosterone is mainly used as therapy for male hypogonadism, delayed puberty, and impotence [28]. In sports, testosterone is used in high doses, with the ability to increase muscle mass [9], strength [9], combat low energy [1], and weight loss [9] all dose-dependent [20]. Exogenous testosterone administration is also linked to various hormonal pathologies, such as the suppression of the hypothalamic–pituitary–adrenal axis function [9], decreasing endogenous testosterone production [51], and leading to testicular atrophy [1,9], erectile dysfunction, and gynecomastia [1,9,20]. The use of testosterone in sports practice is common concomitantly with clomiphene and growth hormone, which induce endogenous testosterone production, reducing testicular atrophy [9,20]; combined with tamoxifen, an anti-estrogen substance, to block gynecomastia symptoms [9,20], and with sildenafil or tadalafil (phosphodiesterase type 5 inhibitors) to erectile dysfunction treatment [9], and finasteride (5-alpha reductase inhibitors) for treating alopecia [9]. Kamran et al. analyzed several football players, divided into three groups, respectively, with complex training, contrast training, and control [60], and concluded that the complex training group performed better performance results in conjunction with higher serum testosterone values (28%), when compared to the contrast training group (17%) [60], and with the control group, which did not show any variation [60].

Thyroid hormones are used for the primary and secondary treatment of hypothyroidism; in sports, the substance is used to combat fatigue, obesity, treat depression, cognitive and physical deficiency, and infertility [66]. Intensive use is associated with adverse effects such as cardiovascular effects (heart failure and tachycardia), iatrogenic thyrotoxicosis, tremors, physical weakness, and alopecia [1].

In addition to hormonal substances, there are others such as diuretics and β2 agonists used to improve physical appearance and control excessive use of hormonal substances. The use of furosemide, a diuretic, is used to treat hypertension [16], chronic renal failure [16], and heart failure [16]. In sports practice, it is mainly sought in weight-category sports as it helps to rapidly and reversibly reduce body weight by decreasing body water [9,19,38] and alters the normal urinary excretion of metabolites of other concomitantly used substances, increasing urine volume and diluting them, making detection more problematic in control tests [56].

Glucocorticoids are commonly prescribed for the treatment of adrenal insufficiency [1], various inflammatory diseases, and as immunosuppressive agents [14]. Due to its potent anti-inflammatory agents that help reduce inflammation in the airways, making them a cornerstone in the management of chronic asthma [14]. In sports practice, glucocorticoids are used off-label to increase respiratory capacity and acute injuries and to manage adrenal fatigue due to the chronic state of physical and emotional stress that athletes experience due to intense training, competition, and the pressure to perform [14,70]. Their anti-inflammatory and immunosuppressive properties are particularly valued for reducing pain and inflammation, allowing athletes to continue training and competing despite injuries [70].

The use of anti-estrogens such as tamoxifen is used to treat breast cancer [10,16] but is sought in sports practice because it blocks the symptoms of gynecomastia caused by exogenous testosterone [9,10,16,20] and relieves fluid retention [20].

Finally, clenbuterol, a β2 agonist, is used in therapeutic doses as a bronchodilator for asthma treatment [9]. In sports practice, it is used off-label to increase lean mass [9], accelerate the lipolysis process and weight loss, and increase metabolic rate [19]. Clenbuterol has ergogenic properties more similar to ephedrine or amphetamine [60].

**Table 2:** Non-hormonal substances used, therapeutic use, off-label use, and risks associated with off-label use in sports training.

Substance Name and Anatomical Therapeutic Chemical Code	Therapeutic Use	Off-Label Use in Sports Training	Previous Described AEs in Off-Label Use
Furosemide (Diuretics) C03CA01	- Hypertension [16]; - Heart failure [16]; - Acute renal failure [16].	- Rapid and reversible weight reduction [38,56] (decrease in body water [9,19,38], providing advantages in weight-category sports [9,56]; - Alter normal urinary excretion of metabolites of other substances used previously (increasing urine volume, diluting them, making detection more problematic in control tests [56].	Dehydration [56]; electrolyte imbalance [18,56]; cramps [56]; dizziness [56]; gastric diseases [18,56]; skin irritation [56]; impotence [56,68].
Clenbuterol (β2 agonists) R03AC14	- Bronchodilator for asthma treatment [17].	- Increase lean mass [9]; - Accelerate the lipolysis process and weight loss [18]; - Increase metabolic rate [19].	Tremors [71]; gastrointestinal disorders [9]; anxiety [9]; severe hypokalemia [71]; hospitalization due to tachycardia [9,17]; death [17].
Glucocorticoids H02AB	- Adrenal insufficiency [1]; - Inflammatory diseases [14]; - Immunosuppressive agent [14]; - Control asthma [14].	- Acute injuries [14,70]; - Adrenal Fatigue [1]; - Increase respiratory capacity [70,72].	Inhibition of the hypothalamic–pituitary–adrenal axis [70]; increased risk of infections; insulin resistance [70]; hypertension and atherosclerosis [73]; osteoporosis [1].
Tamoxifen L02BA01	- Breast cancer treatment [10,16].	- Block symptoms of gynecomastia caused by exogenous testosterone [9,10,16,20]; - Relieve fluid retention [20].	Joint pain [56]; cardiovascular disorder [74]; blood clotting [56]; nausea [56]; blurred vision [56]; pulmonary embolism [74].

## 4. Discussion

### 4.1. Athletes’ Motivations for Off-Label Drug Use

Athletes of varying competitive levels, from elite professionals to recreational participants, face societal pressures that often lead them toward off-label drug use [1]. These pressures include the pursuit of an ideal body image, weight management, and muscle mass increases, alongside attempts to reduce aging signs and manage fatigue or hormonal imbalances [1,10]. One of the primary findings in the literature is that male athletes are generally more inclined to engage in doping behaviors compared to their female counterparts. For instance, studies have shown that males are more likely to use performance-enhancing substances, often motivated by a desire to enhance physical performance and competitive edge, while females tend to use dietary supplements primarily for health-related reasons [75,76]. While the specific motivations may differ between professional athletes seeking a competitive edge and amateur athletes focusing on appearance or fitness, the associated risks remain equally significant [1,10,16]. This use is sometimes based on information from unreliable websites, advertisements, and commercials [1].

Recovery acceleration is another major reason why athletes use off-label medicines. Medications intended for muscle-wasting diseases, such as anabolic steroids, may be used to speed up recovery from injuries or strenuous workouts, allowing athletes to maintain rigorous training schedules [77,78]. The desire to manage weight, especially in sports with strict weight classes for competition or esthetic demands, can lead athletes to use substances for appetite suppression or metabolic enhancement [77]. Diuretics and other medications not primarily intended for weight loss might be used off-label to rapidly lose or control weight [18]. Athletes commonly use substances to enhance physical appearance, lose weight, increase muscle mass, delay aging, and boost performance [1,10,19]. The pressures of society and the media in obtaining an ideal body image [79], together with the desire for quick and often unrealistic results, drive athletes to use these substances, despite the associated health risks and ethical concerns [9]. The desire for improved physical appearance, increased strength, and enhanced recovery can lead athletes to rationalize the use of PED substances, despite the known health risks associated with such practices [38].

The increasing emphasis on body esthetics and ideal athletic performance, especially amplified by social media and contemporary cultural narratives, appears to significantly drive athletes toward off-label drug use. This observation underscores the need for more research to understand the cumulative impact of these sociocultural pressures on the physical and mental health of athletes, particularly among younger and impressionable demographic groups.

### 4.2. Misinformation in Off-Label Use

General consumers tend to seek information through the internet, often obtaining it from unreliable websites, fake advertisements, and misleading social media posts, which can lead to the spread of inaccurate or harmful advice and encourage unsafe practices. [1]. However, much of the available information comes from sources that often prioritize sales over credible medical guidance. This is particularly concerning in athletes, who use hormonal treatments, where inconsistent guidelines and prescription patterns can confuse them, leading them to rely on unverified allegations from forums, social media, or word-of-mouth recommendations [21]. Misinformation plays an important role in the increasing off-label use of prescription drugs, as well as in the increasing incidence of adverse events related to those drugs and broader social and economic risks. [8,21]. This reliance on unverified information leads to improper dosing, incorrect usage, and a misunderstanding of the associated risks and side effects [8]. The lack of regulation and oversight leads athletes to obtain these substances from unreliable sources, including the black market, resulting in the use of counterfeit or contaminated products [20,21]. This misuse has broader social and economic impacts, including increased healthcare costs, social stigmatization, and potential legal consequences [80]. Additionally, athletes often rely on non-credible sources for information, leading to misuse and misunderstanding of the associated risks. The use of PEDs also raises ethical concerns related to fairness in sports and the pressure on athletes to conform to drug use to remain competitive [81].

The accessibility of both reliable and unreliable health information via the internet and social media highlights a crucial gap in health literacy among athletes. Addressing this gap may require stricter regulations on the dissemination of medical content, especially on platforms popular among younger or amateur athletes, who may be more vulnerable to misinformation. Future studies should evaluate the impact of misinformation on off-label drug use practices and explore interventions to enhance health literacy.

### 4.3. Physical, Psychological, and Social Risks of Off-Label Drug Use

The trend of use of off-label by athletes is driven by a variety of psychological, social, and physical factors, motivated by the desire to conform to societal expectations and achieve a more esthetic body, losing weight, increasing muscle mass, reducing signs of aging, and improving performance [1,10,16].

Physically, the use of drugs like anabolic steroids, growth hormones, and erythropoietin is associated with cardiovascular problems, such as hypertension, heart attacks, and the occurrence of stroke [82,83]. Infections are also common, particularly when drugs are administered through injection under non-sterile conditions [84]. Furthermore, users may experience hormonal imbalances [85], including conditions like gynecomastia, testicular atrophy, and menstrual irregularities in women, which can lead to infertility [86]. Psychologically, the misuse of these substances often results in physical and psychological dependence, where users feel compelled to continue their use to maintain physical appearance or performance levels [87]. Beyond individual health, the off-label use of prescription drugs in sports and fitness has broader social and economic implications [1,80]. The increased burden on healthcare systems due to the treatment of adverse effects, the potential for social stigmatization, and the legal consequences of illicit drug use are significant concerns [80]. Additionally, the ethical issues surrounding fairness in sports and the pressure on athletes to conform to drug use practices further complicate the landscape [36].

Social changes and aggressive behaviors are also present, leading to isolation, strained relationships, and described social episodes of violence [88]. Users often withdraw from social interactions, prioritizing their physical goals over relationships, leading to their isolation [87]. There is an increased risk of developing an antisocial lifestyle, and the frequency of crimes of violence and weapon offenses is increased once anabolic steroids are consumed for a long period [88]. Abusers of steroids are more prone to be involved in criminal acts, as demonstrated by epidemiological studies, and steroids are sometimes identified as an indirect cause of death [89,90].

While the physical risks associated with off-label drug use are well documented, the psychological and social consequences, including potential isolation and aggressive behaviors, merit further investigation. These findings suggest that prevention strategies should address not only physiological harm but also provide comprehensive mental health support and counseling for athletes facing dependency or psychological repercussions of such practices.

### 4.4. Economic Consequences

A major concern is the source of these substances; despite the requirement of medical prescriptions for these drugs, athletes often seek alternative sources. Another health risk factor is related to the source for obtaining those substances [1], including online sellers, gym members, or trainers, or even with an inappropriate prescription from a doctor who issues the prescription without the legitimate need or medical indication, simply because it is requested [18]. The rise in smuggling and counterfeiting of pharmaceuticals, particularly through the black market and clandestine laboratories, underscores the critical importance of regulations like the Falsified Medicines Directive (FMD), which aims to protect public health by preventing the distribution of counterfeit medicines within the European Union [18]. This directive is a significant legislative measure designed to secure the pharmaceutical supply chain from illegal and dangerous substances that often enter through illicit means, including black market operations from foreign countries [20,23]. The FMD was introduced by the European Union (EU) in 2011 to combat the distribution of falsified medicines and requires pharmaceutical manufacturers to implement stringent packaging measures such as unique barcodes and tamper-evident seals [32]. Additionally, the directive obligates pharmaceutical stakeholders to verify the authenticity of medicines before they reach consumers, ensuring that medications distributed in the EU are legitimate and safe [32]. Operation Pangea, an annual global crackdown coordinated by INTERPOL, reinforces the necessity of the FMD [91]. This operation specifically targets the illicit online sale of counterfeit and unlicensed medicines. The 2019 Operation Pangea, for instance, resulted in the seizure of 36 million potentially dangerous pills, worth more than $14 million [91,92]. This illustrates the scale of the problem and the international efforts required to tackle it effectively.

Regarding the economic component, off-label use can represent a significant portion of health expenditure [7,93] for the treatment of adverse effects associated with the use. [81]. Sivalokanathan et al. explored the cardiac effects of PEDs, comparing substances like caffeine and anabolic steroids, and emphasized the health risks associated with PEDs that can lead to increased healthcare costs [94]. Off-label drug use places a heavy burden on healthcare systems due to the need for treatment of the various adverse effects [95,96]. Sampaio et al. show that the yearly costs of this off-label use, including complications, among 18-year-old males in Sweden amount to nearly half a million USD [97]. These costs include healthcare expenses, productivity losses, and legal system expenses. Specifically, healthcare costs represent 54% of the total, with productivity losses (28%) and judicial costs (18%) also contributing significantly [96]. The economic impact of off-label drug use, with increased healthcare costs and regulatory challenges regarding counterfeit drug markets, underscores the urgent need for more robust international regulatory collaboration.

### 4.5. Future Research and Interventions

Future research should focus on long-term health effects to understand the consequences of off-label drug use in athletes, particularly across different demographics and types of sports [14]. Effective interventions need to be explored, including educational programs, policy changes, and support systems for athletes [97]. Sampaio et al. suggest that preventive interventions, particularly those targeting gym-goers, can be cost-effective and beneficial from a societal perspective [96].

Another focus must be to investigate the sources from which athletes obtain information about off-label drug use and how to improve the reliability and accessibility of accurate information. Additionally, studies should assess the effectiveness of current regulations and propose new frameworks to better control the distribution and use of these substances in sports [81]. Understanding the psychological motivations behind off-label drug use, such as body image issues, performance pressure, and social influences, is important for developing targeted psychological interventions [81,98]. Comparative studies should also be conducted to compare the prevalence and impact of off-label drug use in different sports, regions, and levels of competition to tailor interventions accordingly. Future research should address the psychological motivators behind off-label drug use among athletes, focusing on young athletes who may be particularly influenced by social pressures. Educational interventions should be tailored, not only for athletes but also for their coaches and support networks, aiming to promote a preventive approach that considers psychological, social, and physical dimensions.

## 5. Conclusions

This review identified key substances commonly used off-label in sports, emphasizing their off-label applications and associated adverse effects. Hormonal (e.g., erythropoietin, insulin, human chorionic gonadotropin, human growth hormone, oxandrolone, testosterone, and thyroid hormones) and non-hormonal (e.g., furosemide, clenbuterol, glucocorticoids, and tamoxifen) are often used by athletes to enhance performance, increase muscle mass, and expedite recovery. However, the off-label use of these drugs carries significant risks, including cardiovascular issues, hormonal imbalances, psychological disturbances, dependency, and severe adverse events like organ damage and death. The data highlights a growing problem, stressing the need for stricter regulations and preventive measures. The off-label use of these drugs poses significant risks and requires targeted interventions to protect athletes’ health and integrity.

## Data Availability

Requests to access the datasets should be directed to the corresponding author and will be granted upon reasonable request.
